# The interaction between selenium and other elements in soil and rice roots shaped by straw and straw biochar regulated the enrichment of selenium in rice grain

**DOI:** 10.3389/fpls.2024.1387460

**Published:** 2024-09-20

**Authors:** Qinlei Rong, Jie Chen, Yufang Zhang, Zebin Tan, Wanjing Wang, Chunxia Sun, Xi Guo, Chunhuo Zhou, Haisheng Cai, Xiaomin Zhao

**Affiliations:** ^1^ Key Laboratory of Crop Physiology, Ecology and Genetic Breeding, Ministry of Education, Nanchang, China; ^2^ College of Land Resources and Environment, Jiangxi Agricultural University, Nanchang, China; ^3^ Key Laboratory of Agricultural Resources and Ecology in Poyang Lake Watershed of Ministry of Agriculture and Rural Affairs in China, Ministry of Agriculture and Rural Affairs, Nanchang, China

**Keywords:** soil Se forms, Se-enriched rice, interaction between Se and other elements, Se-rich paddy fields, straw biochar

## Abstract

The absorption and transport of selenium (Se) in rice depend on the shared transport proteins and channels with other elements. However, the interactions between Se and other elements within the soil–rice system and their relationship with Se-enriched rice are still not well understood. Hence, we conducted pot experiments to investigate the transformation of Se forms in soil and the absorption and enrichment of Se in rice, which varied with other elements influenced by straw and straw biochar returning in Se-rich red paddy soil. Partial least squares path modeling (PLS-PM) analysis was carried out to reveal the interaction between Se and other elements and the crucial processes in Se enrichment in rice grains. The results showed that the incorporation of straw and straw biochar into the fields increased the content of soil-soluble Se (SOL-Se) but significantly decreased the content of iron-manganese oxide-bound Se (FMO-Se) and organic matter-bound Se (OM-Se). Moreover, the rise in the soil-bioavailable Se was mainly attributed to the activation of FMO-Se and OM-Se. Compared with the NPK treatment, the contents of Se in rice grain were increased by 69.22% and 38.09%, under straw and biochar returning, respectively. However, the contents of Se in the leaves decreased. Variation partitioning analysis (VPA) indicated that the migration of Se in rice plants was significantly influenced by differences in rice tissues and their interactions with other nutrients [nitrogen (N), phosphorus (P), potassium (K), and Se], explaining 51.5% and 35.3% of the variations in Se content in different rice parts, respectively. The PLS-PM analysis demonstrated that the absorption of Se by rice roots and its transportation from the leaves to grains were crucial processes affecting Se enrichment in rice. However, these processes were modulated by the interaction between soil properties and root nutrients (N, P, and Se) induced by straw and straw biochar incorporation. The present study provides further understanding of the main factors and key processes in regulating Se absorption and transformation in the soil–rice system to more efficiently utilize Se-rich paddy fields through agricultural management measures.

## Introduction

Selenium (Se) is a crucial trace element for animals and humans, and it also serves as a beneficial element for plants (Zhang et al., 2019; Zhang and Chu, 2022). It has been reported to improve antioxidant activity, enhance immunity, and delay aging (Rayman, 2000; Wang et al., 2022a). An inadequate supply of Se can lead to the development of Keshan disease and Kashin–Beck disease (Tan et al., 2002). Actually, with approximately one billion people suffering from Se deficiency around the world (Chen et al., 2020), it is still a worldwide concern to end hidden hunger as stated in the Sustainable Development Goals (SDGs) (Gödecke et al., 2018). Given that rice is a staple food for a significant portion of the global population, it is an ideal means of enhancing human Se intake through the consumption of staple rice (Rayman et al., 2008). The efficient utilization of valuable Se-rich soil can achieve Se nutrition biofortification of rice, providing a valuable solution to human Se deficiency. However, the Se content in the rice grown in Se-rich paddy fields often fails to meet the Se enrichment standards. Therefore, revealing the factors affecting the Se bioavailability in seleniferous paddy soil and its transport in rice is crucial for achieving Se-rich rice.

Soil serves as the main reservoir of Se for plant uptake, and the diverse distribution of Se forms in the soil is a crucial factor affecting the absorption of Se by plants (Ma et al., 2023). The bioavailability of Se in the soil is regulated by the forms of Se and soil components (Chang et al., 2019). Based on the solubility of different Se forms in the soil solution and their binding capacity with different soil components, there are five forms of Se with gradually decreasing bioavailability: soluble Se (SOL-Se), exchangeable and carbonate-bound Se (EXC-Se), iron-manganese oxide-bound Se (FMO-Se), organic matter-bound Se (OM-Se), and residual Se (RES-Se) (Wang et al., 2012). The differential distribution of Se forms in the soil reflects the bioavailability of Se. Although the total Se content is relatively high, the proportion of bioavailable Se forms, which are more effective for plants, is low (Ma et al., 2023). Studies indicate that in Se-rich soils, Se mainly exists in the forms of organic-bound Se and residual Se, with a lower content of bioavailable Se (Xiao et al., 2020). Furthermore, the transformation of Se forms in the soil is closely related to the nutrient status of the soil, including the content of soil organic matter, nitrogen, phosphorus, and potassium (Pang et al., 2022; Wang et al., 2022b). Straw and straw biochar returning, rich in carbon, nitrogen, phosphorus, potassium, and trace elements, is considered to be a common and effective measure to improve soil fertility (Islam et al., 2022; Bagheri Novair et al., 2023). As exogenous nutrients are brought into the soil, it will inevitably change the transformation of soil nutrients (Singh et al., 2022; Liu et al., 2023), thus affecting the distribution of selenium in the soil. However, the impact of returning straw and straw biochar on the transformation of Se forms in Se-rich paddy soils is not yet clear.

Plants primarily absorb Se in the forms of selenate (VI), selenite (IV), and organic Se [selenocysteine (SeCys) and selenomethionine (SeMet)], but they generally do not uptake Se(0) and Se(-II) (White, 2016; Chauhan et al., 2019). As specific Se transport has not yet been found in plants (Zhou et al., 2020), the uptake, transport, and subcellular distribution of Se mainly rely on transport proteins and channels associated with other elements (Zhang and Chu, 2022). In red paddy soil, selenite (SeO_3_^2−^, HSeO_3_^−^, H_2_SeO_3_) is the most abundant water-soluble form (White, 2018). Research indicates that different forms of selenite (SeO_3_^2−^, HSeO_3_^−^, H_2_SeO_3_) have distinct absorption mechanisms. At a pH of 5, Se in the form of HSeO_3_^−^, with similar ionic radii and comparable physical and chemical properties to phosphate, is primarily absorbed through phosphate transporters (Zhang et al., 2014). It has been reported that there was either an antagonistic or synergistic relationship between phosphorus and Se in crops, which may be influenced by the concentration of phosphorus and Se and soil conditions (Hu et al., 2023; Huang et al., 2024). Under more acidic conditions, Se in the form of H_2_SeO_3_ is mainly absorbed by rice through aquaporins and/or a silicon influx transporter (Zhang et al., 2010; Zhao et al., 2010). Conversely, at higher pH levels, selenite in the form of SeO_3_^2−^ is absorbed by rice, which exhibits similar absorption and transport pathways of sulfur in plants (González-Morales et al., 2017). After plants absorb selenite, it undergoes a transformation into organic Se, such as selenomethionine (SeMet), before being transported to the shoots (Yu et al., 2019). So, it can be calculated that the absorption and translocation of Se by rice are closely related to nutrient elements such as nitrogen, phosphorus, and sulfur. Activating Se in the soil through agronomic measures and promoting its transfer within the plant are practical and feasible methods for achieving Se enrichment in rice in a green and simple manner. However, gaining a more profound insight into the pivotal coupling elements and processes associated with the absorption and translocation of selenium in the soil–rice system is essential.

Enhancing the bioavailability of Se in Se-rich soils and regulating the transfer of Se from rice roots to grains are beneficial for Se enrichment in rice grains. Therefore, the main objectives of this study are 1) to investigate the influence of straw and straw biochar on the transformation of Se forms in red paddy soils, 2) to examine the absorption and transportation characteristics of Se in rice with the application of straw and straw biochar, and 3) to analyze the influencing factors of Se enrichment in rice grains in the soil–rice system. Studying the impact of straw and straw biochar on the absorption and accumulation of Se in rice would provide valuable insights for the regulation of Se absorption in rice through agricultural management measures.

## Materials and methods

### Experimental soil

According to the field survey results of our previous study (Sun et al., 2023), the seleniferous paddy soil was sampled from the plow layer (0–20 cm) of red paddy fields in Yuanzhou District, Jiangxi, China, with coordinates of 27.72°N and 114.18°E. The soil was air-dried and then sieved through a 2-mm mesh for the pot experiment. The soil pH value was 6.90; soil organic matter (SOM) was 47.58 g/kg; total nitrogen (TN) was 1.37 g/kg; the alkaline hydrolysis nitrogen (AN) was 43.83 mg/kg; the available phosphorus (AP) was 13.54 mg/kg; the available potassium (AK) was 54.90 mg/kg; and total Se concentration was 0.47 mg/kg.

### Pot experiment design

A pot experiment was conducted to evaluate the effects of straw and straw biochar on soil properties, Se transformation, and the uptake and transport of Se by rice in Se-enriched paddy soil. The tested rice variety is Taiyou 398. Three treatments were established in the pot experiment, namely, 1) the control treatment (NPK, only chemical fertilizers), 2) the straw treatment (NPK+S, chemical fertilizers + 1% (m/m) addition of straw), and 3) the straw biochar treatment (NPK+B, chemical fertilizers + 1% (m/m) addition of straw biochar). A completely randomized experimental design was adopted, with three treatments, three replicates per treatment, and a total of nine pots. To ensure consistent lighting conditions, the pots were repositioned randomly every week to mitigate the impact of the microenvironment.

The specifications for the pots used in the pot experiment were approximately 25 cm in height, 18 cm in bottom diameter, and 23 cm in top diameter. Each pot contained 7.5 kg of soil. Equal amounts of nitrogen (N), phosphorus (P), and potassium (K) chemical fertilizers were applied in each treatment, with pure N at 0.15 g/kg, P_2_O_5_ at 0.10 g/kg, and K_2_O at 0.15 g/kg. The biochar used in the pot experiment was prepared by pyrolysis of the rice straw at 550°C for 2 h in a muffle furnace. The tested fertilizers were urea (46% N), calcium magnesium phosphate (12% P_2_O_5_), and potassium chloride (60% K_2_O). The total nitrogen of the straw was 11.76 g/kg; the total phosphorus was 1.96 mg/kg; the total potassium was 15.29 mg/kg; and the total Se was 0.08 mg/kg. Moreover, the total nitrogen of the straw biochar was 12.33 g/kg; the total phosphorus was 8.60 mg/kg; the total potassium was 47.71 mg/kg; and the total Se was 0.06 mg/kg. Both the added straw and straw biochar, as well as chemical fertilizers, were applied as basal fertilizer at once. After a week of water saturation equilibrium, rice seedlings were transplanted. Each pot was planted with three holes, and two seedlings with uniform growth in each hole were selected for transplantation. Other management measures followed field production practices.

### Sampling

During the rice maturation stage, the plant and soil samples were collected on 25 October 2022. The soil samples of the pot experiment were uniformly collected in the pot by the multipoint sampling method and then mixed evenly and transported to the laboratory for further processing in a self-sealing bag. Non-soil components, such as plant roots, were removed from the soil samples, which were then naturally air-dried. The air-dried soil samples were then ground and sieved through 20-mesh and 100-mesh sieves before being packed into self-sealing bags for subsequent determination of soil physicochemical properties, total Se content, and Se forms.

After removing the entire rice plants from the pots, the soil adhering to the roots was carefully detached, taking care not to damage the rice roots. The plants were then brought to the laboratory for processing. The rice roots were rinsed with water to clean them from soil particles. Clean absorbent paper was used to blot excess moisture from the root surfaces. The rice plants were divided into several parts: roots, stems, leaves, and grains, each of which was placed in separate paper bags. These bags were placed in an oven at 105°C for 30 min to halt biological activity, and then the oven temperature was adjusted to 65°C to continue drying the plant samples until a constant weight was achieved. The dry biomass accumulation of each plant part was determined using a 1% balance. Samples of different plant parts were ground using a grinder and stored in self-sealing bags for subsequent analysis of plant nutrient content and Se concentration.

### Analysis of soil and plant samples

Soil physicochemical properties were analyzed according to Bao (2000). Soil pH was determined using potentiometry with a mixture of soil and water at a ratio of 1:2.5. An elemental analyzer (Vario MACRO cube, Elementar, Germany) was employed to measure the contents of SOM and total N. The AN content was determined by the alkali diffusion method. The AP content was determined using the Olsen method. The AK was extracted with a 1-mol/L NH_4_AC solution by an atomic absorption spectrometer (contrAA 700, Germany). After digested using a concentrated sulfuric acid and hydrogen peroxide mixture, the concentrations of N, P, and K in different parts of the rice plant were determined by the Kjeldahl method, vanadium molybdate colorimetric method, and flame photometry, respectively.

Soil samples were digested with 3:2 (v/v) HNO_3_/HClO_4_ and then reduced to Se(IV) in 6.0 mol/L of HCl solution (extracts:solution, 1:1) at 100°C for 30 min, following the method reported by Ma et al. (2023). Plant samples were digested with 9:1 (v/v) HNO_3_/HClO_4_ via microwave digestion. After reduction with hydrochloric acid, the Se content was determined by an atomic fluorescence spectrophotometer (XGY-1011A, Institute of Geophysics and Geochemistry Exploration, CAGS, China) with hydride generation according to the China National standard method (GB 5009.93-2010). The extraction of Se fractionations in soils (SOL-Se, EXC-Se, FMO-Se, OM-Se, and RES-Se) was conducted using the sequential continuous extraction method (Wang et al., 2012).

### Statistical analysis

Data organization and cleaning were conducted using Excel 2016 (Microsoft Corp., Redmond, WA, USA). Graphs were generated using SigmaPlot 12.0 (Systat Software Inc., USA). Variance analysis utilized the completely randomized analysis method to compare the significance among different treatment variables (LSD, *p* = 0.05). Pearson correlation analysis was employed using the *Hmisc* package in R (v.4.1.1) to analyze the correlation between soil chemical properties, soil total Se, soil Se forms, and the relationship between plant nutrients and plant Se content. Variation partitioning analysis (VPA) was performed using the *vegan* package in R (v.4.1.1) to quantify the significance of different parts of rice and plant nutrients on the variation in Se content in different parts of rice. Partial least squares path modeling (PLS-PM) analysis was carried out using the *plspm* package in R (v.4.1.1) to reveal the relationships between soil chemical properties and plant nutrients (N, P, K, Se) and the Se enrichment coefficient in rice.

The calculation of Se accumulation in different parts of the plant was represented by Equation 1:


(1) $${\text{Se}}_{\text{accum}\;i}={\text{C}}_{i}\times B_{i}$$

Se_accum_ *_i_* represented the Se accumulation in the *i*th part of rice (mg). *C_i_* represented the Se content in the *i*th part of rice (mg/kg). *B_i_* represented the dry mass of the *i*th part of rice (g). Here, *i* can take on values representing different parts of the rice plant, such as the roots, stems, leaves, and grains.

The enrichment factor of Se in rice has been calculated using the following Equation 2:


(2) $${\text{BCF}}_{\text{Rice}}=\frac{{\text{C}}_{\text{Rice}}}{{\text{C}}_{\text{Soil}}}$$

In the equation, BCF_Rice_ represented the bioconcentration factor of Se in rice grains. C_Rice_ represented the Se content in rice grains (mg/kg). C_Soil_ represented the total Se content in the soil (mg/kg).

The transfer coefficient of Se in different parts of the plant was calculated according to the following Equation 3:


(3) $${\text{TF}}_{\text{i}/\text{j}}=\frac{{\text{C}}_{\text{i}}}{{\text{C}}_{\text{j}}}$$

The term TF*_i/j_* represented the transfer coefficient for Se moving from one part of the rice plant (*j*) to another part (*i*). In this equation, C*_i_* represented the Se content in part *i* of the rice plant (mg/kg), and C*_j_* represented the Se content in part j of the rice plant (mg/kg). This equation could be used to quantify the transfer of Se between different parts of the rice plant, providing insights into how Se was distributed and transported within the plant.

## Result

### Soil chemical properties of different treatments

As shown in **Table 1**, it can be observed that the NPK+S and NPK+B treatments significantly influenced soil chemical properties. When compared to the treatment of applying chemical fertilizers alone (NPK), the soil pH increased significantly in the treatments of straw return (NPK+S) and straw biochar return (NPK+B), with an average increase of 1.60% and 3.03%, respectively. Furthermore, the NPK+S and NPK+B treatments resulted in significantly higher levels of TN, AN, and AK in the soil compared to the NPK treatment (*p* < 0.05), with increases ranging from 9.83% to 16.08% for TN, 17.14% to 24.33% for AN, and 196.40% to 519.77% for AK. Regarding the impact on SOM and AP, the NPK+B treatment significantly increased these parameters by an average of 35.61% and 23.71% (*p* < 0.05), respectively, compared to the NPK treatment. However, there were no significant differences when compared to the NPK+S treatment.

**Table 1:** Effects of straw and straw biochar returning on soil chemical properties.

Treatment	pH	SOM (g/kg)	TN (g/kg)	Alkaline hydrolyzable N (mg/kg)	Alkaline P (mg/kg)	Available K (mg/kg)
NPK	6.98 ± 0.09b	46.40 ± 4.51b	1.43 ± 0.09b	95.96 ± 8.50b	9.66 ± 0.53b	62.03 ± 4.62c
NPK+S	7.09 ± 0.09ab	54.62 ± 2.64ab	1.66 ± 0.04a	119.31 ± 7.26a	10.74 ± 0.74ab	183.84 ± 6.77b
NPK+B	7.19 ± 0.13a	62.92 ± 6.31a	1.57 ± 0.10a	112.41 ± 5.20a	11.95 ± 1.19a	384.42 ± 22.98a

Different letters in the same column in the table indicate significant differences between treatments (p < 0.05).

### Changes of total Se in soil under different treatments

**Figure 1:**
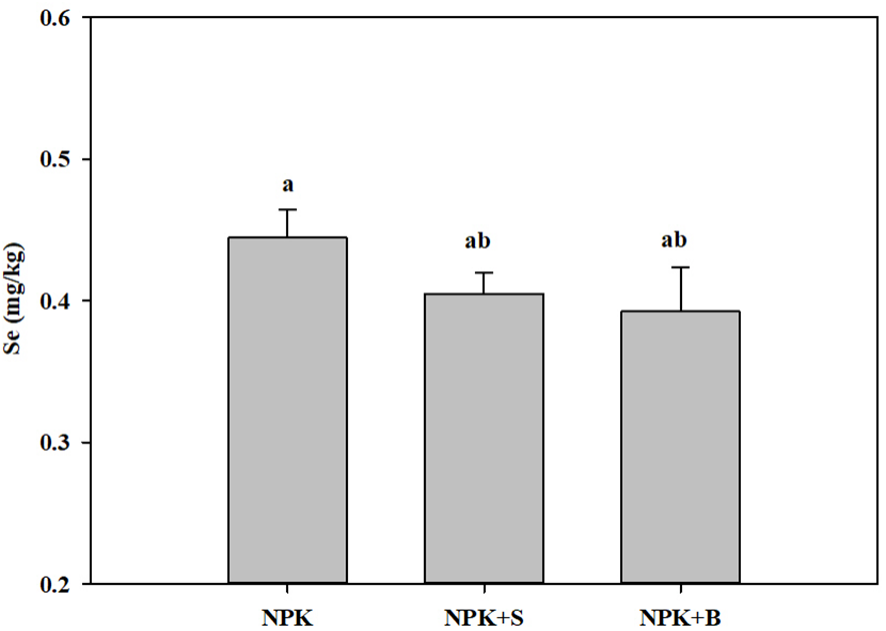
Effects of straw and straw biochar returning on soil total Se content. The bars represent mean ± SE. Significant differences among treatments are denoted with different lowercase letters at the 0.05 level.

The changes in soil Se content under different fertilization treatments are illustrated in **Figure 1**. Among all the fertilization treatments, the soil Se content was the highest in the treatment with chemical fertilizers alone, while it was the lowest in the straw biochar incorporation treatment, with an average reduction of 11.71%. There was no significant difference between the straw return treatment and the straw biochar incorporation treatment (*p* > 0.05), but both were lower than the treatment with chemical fertilizers alone.

### Transformation of Se forms in soil under different treatments

**Figure 2:**
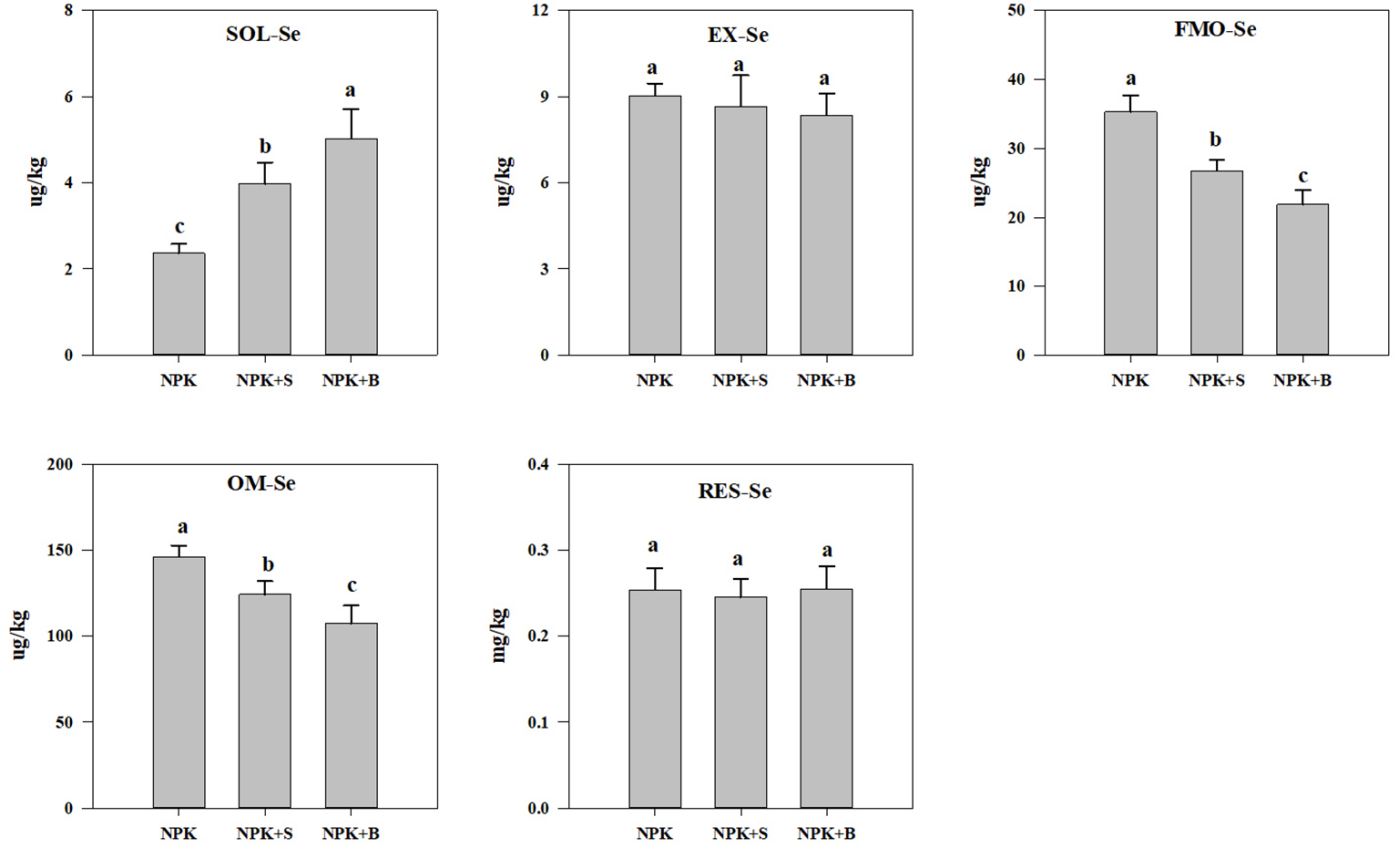
The influence of straw and straw biochar incorporation on different forms of Se content in the soil. SOL-Se, soluble Se; EXC-Se, exchangeable and carbonate-bound Se; FMO-Se, Fe/Mn oxide-bound Se; OM-Se, organic matter-bound Se; RES-Se, residual Se. The bars represent mean ± SE. Significant differences among treatments are denoted with different lowercase letters at the 0.05 level.

Different fertilization treatments significantly altered the distribution of Se speciation in soil. As shown in **Figure 2**, compared to the NPK treatment, the soil SOL-Se significantly increased in the NPK+S and NPK+B treatments (*p* < 0.05), with average increases of 67.72% and 112.09%, respectively. Additionally, the NPK+B treatment showed a significant increase in SOL-Se content compared to the NPK+S treatment. Regarding EXC-Se, there were no significant differences between different fertilization treatments, but the trend in content was NPK > NPK+S > NPK+B. In contrast to SOL-Se, both the FMO-Se and OM-Se contents exhibited a significant decrease in the NPK+S and NPK+B treatments compared to the NPK treatment (*p* < 0.05). The reduction ranged from 24.41% to 38.05% and 15.07% to 26.52%, respectively, with average decreases of 31.23% and 20.79%. Moreover, the NPK+B treatment showed a significant decrease in FMO-Se and OM-Se contents compared to the NPK+S treatment, with average reductions of 18.04% and 13.49%, respectively. However, different fertilization treatments had a relatively small impact on soil RES-Se content, with no significant differences observed between the NPK treatment, NPK+S treatment, and NPK+B treatment (*p* > 0.05). This suggests that straw and straw biochar incorporation primarily affects the SOL-Se, FMO-Se, and OM-Se contents in the soil, while the changes in EXC-Se and RES-Se content are relatively minor.

**Figure 3:**
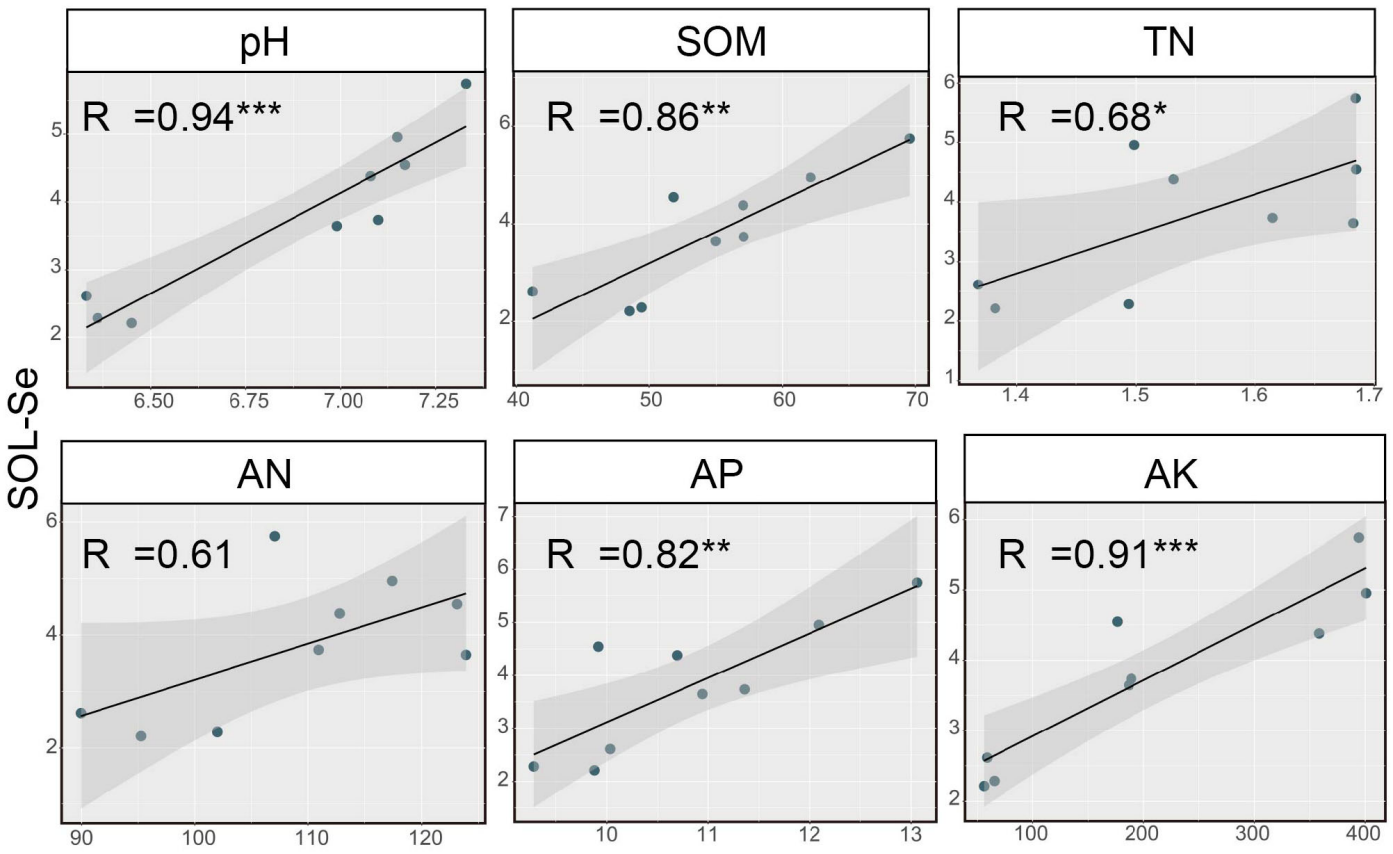
Correlation analysis between soil SOL-Se and soil chemical properties. SOL-Se, soluble Se; SOM, soil organic matter; TN, total nitrogen; AN, alkaline hydrolyzable N; AP, available phosphate; AK, available K. *, **, and *** indicate a significant correlation at the 0.05, 0.01, and 0.001 levels, respectively. The shadow part represents the 95% confidence interval of the fitting curve.

Further analysis revealed a close relationship between soil SOL-Se and soil pH and nutrient status. The correlation analysis between SOL-Se and soil chemical properties is shown in **Figure 3**. SOL-Se showed significant or highly significant positive correlations with soil pH, SOM, TN, AP, and AK (*p* < 0.05), with correlation coefficients of 0.85, 0.86, 0.68, 0.82, and 0.91, respectively. However, the correlation between SOL-Se and AN was not significant, indicating that the effect of AN components on SOL-Se content is relatively small, with a correlation coefficient of 0.61 (*p* > 0.05).

### Rice biomass under different treatments

The impact of straw and straw biochar on the biomass of rice plants is shown in **Table 2**. The results indicated that the NPK+B treatment resulted in the highest total biomass, while the NPK+S treatment had lower biomass compared to the NPK treatment. In particular, the root biomass in the NPK+S treatment decreased significantly by 17.49% compared to the NPK treatment (*p* < 0.05). However, there were no significant differences between the NPK+B treatment and the NPK treatment. Stem biomass did not vary significantly among different treatments. On the other hand, both straw and straw biochar amendments promoted an increase in leaf biomass. Specifically, the NPK+B treatment significantly increased leaf biomass by 36.58% compared to the NPK treatment (*p* < 0.05). Additionally, straw and straw biochar amendments led to an increase in rice grain biomass by 7.42% to 10.01%, with the highest rice grain biomass observed in the straw biochar treatment. Aboveground biomass was increased in both straw and straw biochar treatments, with an increase ranging from 5.77% to 9.49%, although the differences among treatments were not significant.

**Table 2:** Effects of different treatments on dry biomass of rice plants in the mature period (g).

Treatments	Root	Stem	Leaf	Rice grain	Total dry matter weight
NPK	32.30 ± 1.42a	27.68 ± 2.95a	11.68 ± 1.27b	54.43 ± 1.42a	126.09 ± 4.16a
NPK+S	26.65 ± 2.80b	26.78 ± 2.84a	13.96 ± 2.03ab	58.47 ± 3.49a	125.85 ± 5.34a
NPK+B	33.42 ± 3.01a	26.86 ± 2.15a	15.95 ± 1.75a	59.88 ± 4.13a	136.11 ± 8.25a

Different letters in the same column in the table indicate significant differences between treatments (*p* < 0.05).

### The uptake, accumulation, and translocation of Se in rice under different treatments

**Figure 4:**
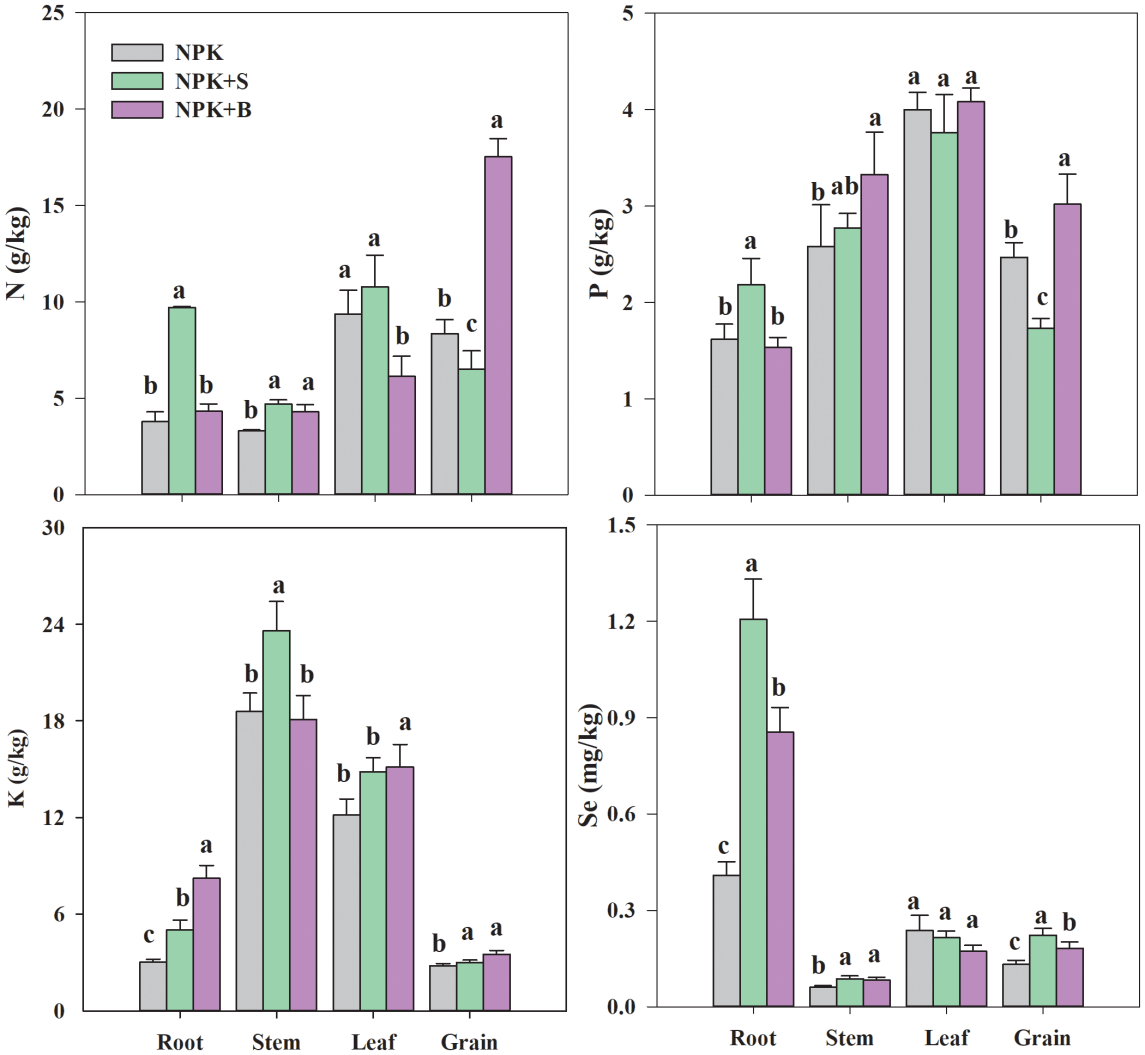
Effects of straw and straw biochar returning on N, P, K, and Se absorption in different parts of rice at the mature stage. Significant differences among treatments are denoted with different lowercase letters in the same part of rice at the 0.05 level.

From **Figure 4**, it could be observed that the NPK+S treatment had the highest N content in the roots, stems, and leaves, with increases of 155.66%, 41.84%, and 15.09%, respectively, compared to the NPK treatment. However, compared to the NPK treatment and the NPK+S treatment, the NPK+B treatment significantly increased the contents of N, P, and K nutrients in the rice plant’s grain (*p* < 0.05), with average increases of 109.65%, 22.28%, and 25.33%, respectively. Compared to the NPK treatment, the straw return treatment increased N content in the roots and stems but decreased P content in the leaves and rice grain (**Figure 4**). Except for the stems, the K content in all parts of the NPK+B treatment was the highest and significantly different from the NPK treatment.

The influence of straw and straw biochar return on Se absorption by rice plants in seleniferous rice fields is shown in **Figure 4**. Overall, except for the NPK treatment, the order of Se content in various parts of rice plants in both the NPK+S and NPK+B treatments was root > rice grain > leaves > stems. There were significant differences in Se content among the treatments in rice roots, with the NPK+S treatment having the highest Se content, followed by the NPK+B treatment, and the NPK treatment having the lowest (**Figure 4**). Compared to the NPK treatment, the NPK+S and NPK+B treatments significantly increased the Se content in the stems and rice grain by 43.75% and 69.22% and by 37.27% and 38.09%, respectively (*p* < 0.05). Straw and straw biochar treatments showed a decreasing trend in Se content in the leaves, with reductions of 9.50% and 27.41%, respectively, but the differences were not significant. Compared to the NPK treatment, both the NPK+S and NPK+B treatments significantly increased the Se content in the rice grain, with the straw return treatment having a higher Se content than the straw biochar treatment.

**Figure 5:**
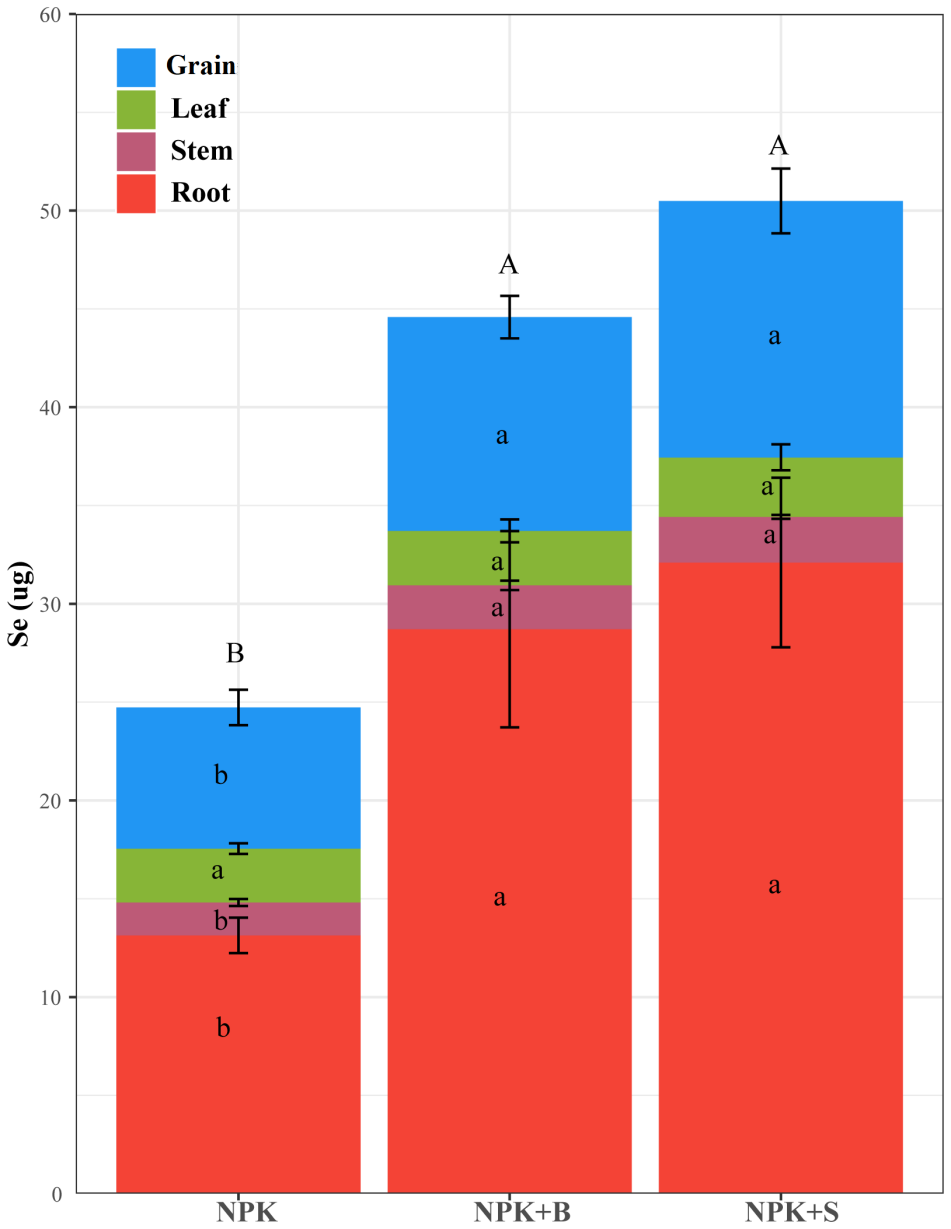
Effects of straw and straw biochar returning on Se accumulation in different parts of rice at the mature stage. Different capital letters on the column indicate that the total Se accumulation is significantly different between treatments at the 0.05 level. Significant differences among treatments are denoted with different lowercase letters in the same part of rice at the 0.05 level.

The changes in Se accumulation in different parts of rice plants in seleniferous rice fields due to straw and straw biochar return treatments are shown in **Figure 5**. It could be observed that both the NPK+S and NPK+B treatments resulted in significantly higher Se accumulation in rice plants compared to the NPK treatment. However, there was no significant difference in the total Se accumulation between these two treatments.

In terms of different plant parts, there were no significant differences in Se accumulation in the leaves among all treatments. However, for the roots, stems, and rice grains, both the NPK+S and NPK+B treatments showed significantly higher Se accumulation compared to the NPK treatment. Specifically, Se accumulation increased by 144.36%, 38.52%, and 81.71% in the roots, stems, and ears for the NPK+S treatment and by 118.50%, 33.27%, and 51.36% for the NPK+B treatment, respectively, compared to the NPK treatment. This indicated that the differences in Se accumulation in rice plants were primarily attributed to variations in the roots, stems, and rice grains.

### Se enrichment in rice grains and their interactions with other elements under different treatments

From **Table 3**, we could observe that the TFs for different parts of rice under different treatments generally exhibit the following order: TF_Leaf/Stem_ > TF_Rice/Stem_ > TF_Root/Soil_ > TF_Rice/Leaf_ > TF_Stem/Root_, with average values of 2.83, 2.31, 2.03, 0.89, and 0.11, respectively. The application of straw and straw biochar significantly increased the TF for root/soil (TF_Root/Soil_) and grain to leaf (TF_Rice/Leaf_). On average, TF_Root/Soil_ increased by 225.51%, and TF_Rice/Leaf_ increased by 137.22%. Specifically, the TF_Root/Soil_ was significantly higher in the NPK+S treatment compared to the NPK+B treatment in the root part although there was no significant difference between them in the rice grain part. In contrast, TF_Leaf/Stem_ and TF_Stem/Root_ were significantly lower in the NPK+S and NPK+B treatments compared to the NPK treatment. In the NPK+S treatment, Se had the highest TF from stems to grains, significantly higher than in the NPK+B treatment and NPK treatment. However, in the NPK+B treatment, the TF from stems to grains did not significantly increase compared to the NPK treatment. Further analysis of BCF_Rice_ in rice revealed that different treatments followed the order of NPK+S treatment > NPK+B treatment > NPK treatment, with significant differences among them. The higher BCF_Rice_ in the NPK+S treatment compared to the NPK+B treatment was mainly due to the NPK+S treatment promoting the transfer of Se from soils to roots and leaves to grains.

**Table 3:** Effects of different treatments on selenium transport coefficient and selenium enrichment coefficient of rice at the mature stage.

Treatment	TF_Root/Soil_	TF_Stem/Root_	TF_Leaf/Stem_	TF_Rice/Stem_	TF_Rice/Leaf_	BCF_Rice_
NPK	0.92 ± 0.10c	0.15 ± 0.02a	3.92 ± 0.67a	2.18 ± 0.23b	0.56 ± 0.05b	0.30 ± 0.03c
NPK+S	2.99 ± 0.42a	0.07 ± 0.01b	2.49 ± 0.42b	2.56 ± 0.19a	1.05 ± 0.19a	0.55 ± 0.05a
NPK+B	2.18 ± 0.07b	0.10 ± 0.01b	2.08 ± 0.30b	2.19 ± 0.05b	1.06 ± 0.13a	0.46 ± 0.02b

Different letters in the same column in the table indicate significant differences between treatments (*p* < 0.05).

**Figure 6:**
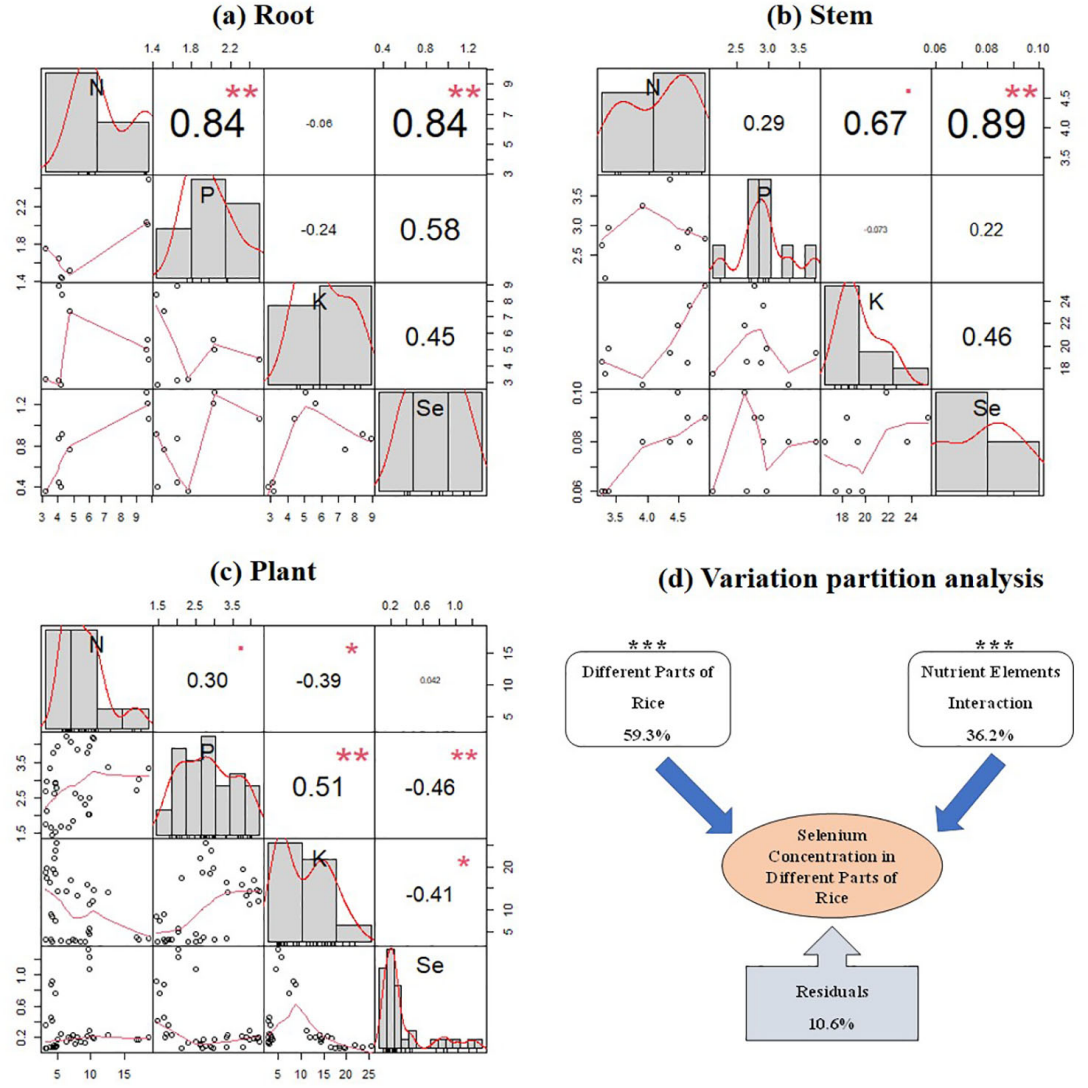
Correlation between the N, P, and K nutrient contents and the Se content at the mature stage in the plant roots **(A)**, stems **(B)**, and the whole rice plant **(C)**, and variation partition analysis (VPA) of the effects of nutrient content changes and different parts of rice on Se content in rice **(D)**. *, **, and *** indicate a significant correlation at the 0.05, 0.01, and 0.001 levels, respectively.

The correlation analysis of the content of N, P, and K nutrients with Se content revealed significant relationships in the roots and stems of rice plants (*p* < 0.05) (**Figures 6A, B**). However, when considering the entire rice plant, there was a significant negative correlation between Se content in different parts of the rice plant and P and K contents (*p* < 0.05) (**Figure 6C**). Furthermore, VPA was conducted to analyze the influence of rice plant parts and their nutrient contents (N, P, and K) on Se content in different parts of rice plants (**Figure 6D**). The results showed that both rice plant parts and their nutrient contents (N, P, and K) significantly affected Se content in different parts of rice plants, explaining 59.3% and 36.2% of the variation in Se content in different parts of rice plants, respectively.

**Figure 7:**
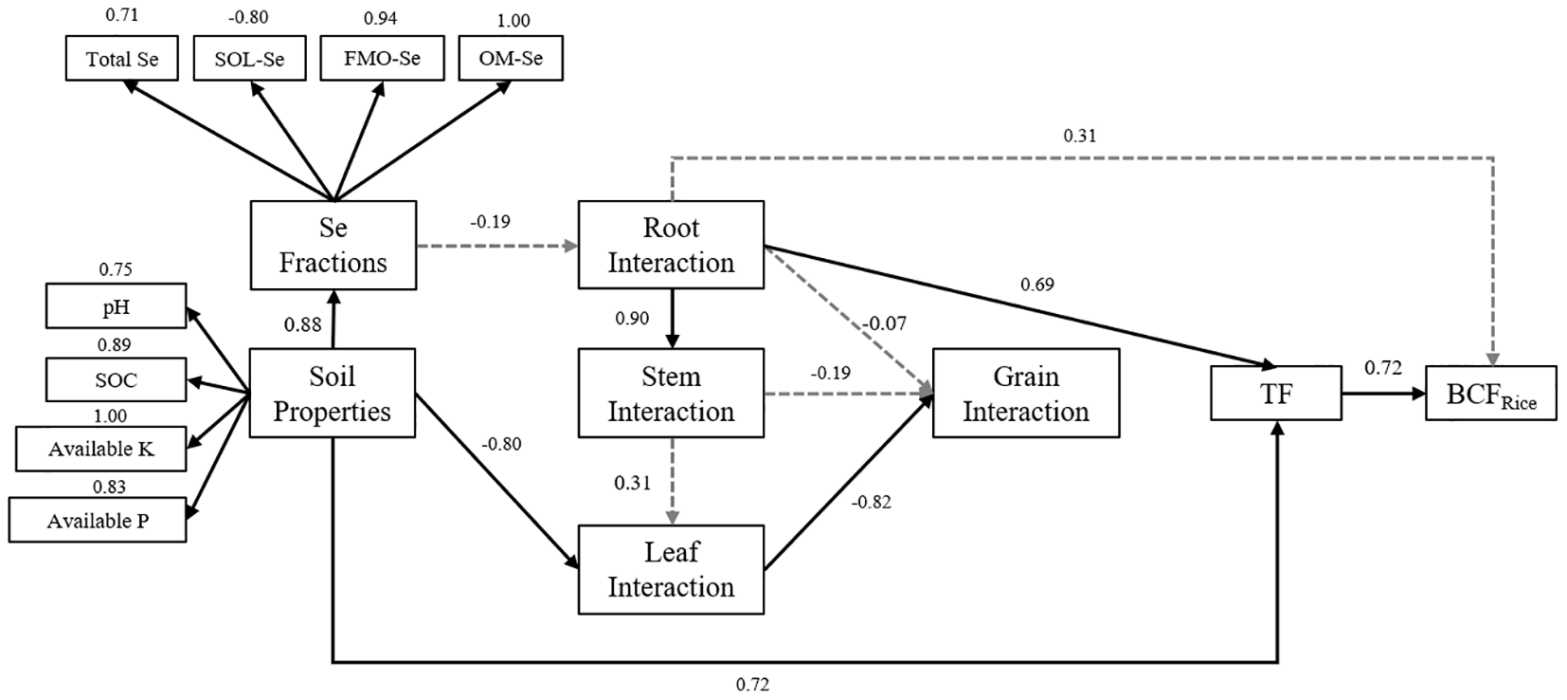
Partial least squares path modeling (PLS-PM) analysis of the soil chemical properties, the interaction of nutrients in different parts of rice, and Se enrichment index under different fertilizer treatments. Path coefficients are calculated after 1,000 bootstraps. The goodness of fit of the model is 0.7297.

The PLS-PM analysis revealed important relationships between soil chemical properties, interactions of nutrients in different parts of rice plants (N, P, K, Se), and the Se enrichment index in rice plants, as depicted in **Figure 7**. The result indicated that the interactions of nutrients in the root part of rice plants significantly influenced the TFs in different plant parts, which in turn significantly affected the BCF_Rice_ of rice plants. In addition, further analysis showed that soil chemical properties, including pH, SOM, AP, and AK (loading values: pH = 0.747, SOM = 0.885, AP = 0.830, AK = 1.000), were identified as the primary soil-related factors influencing the distribution of Se forms in the soil and Se enrichment in rice. The interactions among root nutrients (loading values: N = 1.000, P = 0.841, Se = 0.846) were identified as a crucial plant-related factor influencing the distribution of Se in the rice plant. Specifically, the transfer processes from soil to roots and from leaves to rice grains (loading values: TF_Root/Soil_ = 0.931, TF_Rice/Leaf_ = 0.910) were key processes affecting Se transport within the rice plant. Both soil and plant factors jointly regulated the transport of Se from the soil through the roots, stems, and leaves and ultimately to the rice grains.

## Discussion

### Effects of straw and biochar on the transformation of soil Se forms and its influencing factors

Previous studies have shown that although the interaction between soil physicochemical properties and the content and distribution of Se in the soil is complex (He et al., 2022; Lei et al., 2022), it is a feasible measure to regulate the effectiveness of Se in the soil through changes in soil physicochemical properties within a certain range (Zhang et al., 2023). The results of this study also demonstrated that the application of straw and straw biochar to soil significantly increased the content and proportion of soil SOL-Se (**Figure 2**). Further analysis revealed a significant positive correlation between soil SOL-Se and soil pH, SOM, TN, AP, and AK (**Figure 3**). This is because the negative charge on the surface of soil particles generally increases with an increase in pH, leading to enhanced electrostatic repulsion of selenate and selenite ions with negative charges, releasing Se from the solid phase of soil, thereby increasing the bioavailability of Se (Matos et al., 2017). However, some research indicates that SOM has a dual effect on effective Se. Soil Se can be adsorbed, chelated, or complexed by organic compounds, which reduces the content of soil Se (Wang et al., 2019). On the other hand, with the mineralization of soil organic matter, some organically bound Se in the soil will be converted into small molecule organic Se with higher solubility. The increase in the content of soluble or exchangeable Se due to this transformation can to some extent increase the bioavailability of soil Se (Chang et al., 2019; Dinh et al., 2021). It should be noted that the interaction between soil organic matter and Se is also influenced by soil pH. Organic matter has a higher affinity for Se in acidic soil, and an increase in pH promotes the mineralization of organic Se (Supriatin et al., 2016). Therefore, the combined effect of increased soil pH and soil organic matter due to the application of straw and straw biochar contributes to the increase in soil SOL-Se content.

The first two forms of extraction in the continuous five-step extraction method, generally referred to as soil-effective Se (Wang et al., 2012), include soluble and exchangeable Se. The results of this study indicate that the increase in soil-effective Se was primarily achieved through the transformation of soil Se forms, particularly through the activation of iron and manganese oxide-bound Se and organic-bound Se, which enhances soil-effective Se (**Figure 2**). The organic matter and metal oxides, affected by dry–wet alternation during the growth of rice (Bagheri-Novair et al., 2020), can regulate the complexation, adsorption, desorption process, and valence change of Se, thus affecting the transformation of the soil selenium form (Li et al., 2017; Kushwaha et al., 2022). In this study, the addition of straw and straw biochar reduced the content and proportion of these two forms, likely because the introduction of organic materials promotes the formation of stable organic coordination complexes through the combination of metal oxides with organic matter, reducing the adsorption of Se by iron-aluminum oxides and increasing the effectiveness of soil Se (Dinh et al., 2017). The abundant oxygen-containing functional groups and the high content of exchangeable basic ions and porous structures on the surface of biochar are more favorable for competing with metal oxides for adsorption sites with Se (Zhu et al., 2015), thereby reducing the content and proportion of organic-bound Se and iron-manganese oxide-bound Se. The content and proportion of exchangeable Se in the soil were relatively stable, and the less activatable residual Se underwent a relatively small change, while the activation and migration of other forms of Se caused a significant increase in the proportion of residual Se (**Figure 2**). The total Se content in the soil declined with the application of straw and straw biochar, likely due to the enhanced bioavailability of Se in the soil and the increased uptake and removal by rice after straw and straw biochar application. Additionally, some studies have shown that the input of organic materials can promote the biotransformation of Se (Saghafi et al., 2020), leading to volatilization losses of Se in the soil (Yu et al., 2020). This indicates that even in Se-rich regions, it is important to consider the return and supplementation of Se to achieve sustainable development of the Se industry.

### Effects of straw and biochar on Se enrichment in rice grains and their interactions with other elements

The findings of this study demonstrated that returning straw and straw biochar to the field significantly increased the Se content in the roots and grains (**Figure 5**). This was related to the transformation of soil chemical properties and Se forms induced by returning straw and straw biochar to the field, where a higher content of soil SOL-Se was advantageous for rice absorption (**Figure 2**). The higher soil phosphorus content promotes Se desorption from the soil, thereby increasing the bioavailability of Se in the soil (Huang et al., 2024). In this study, returning straw and straw biochar treatments increased soil pH, enhanced phosphorus nutrient supply, and increased the content of available phosphorus in the soil, all of which were favorable changes for improving Se bioavailability in the soil. The results of Se accumulation and distribution in different parts of rice showed that Se mainly accumulated in the roots of rice plants (**Figures 4**, **5**), consistent with previous research findings (Chang et al., 2019; Shen et al., 2019). Accumulation of higher Se content in the roots of rice indicated that a substantial amount of Se can be retained during the transport of Se from the roots to aboveground tissues (Winkel et al., 2015; Zhou et al., 2020). The selenite absorbed by rice rapidly transforms into organic forms before being transported to the aboveground parts in paddy soil (Yu et al., 2019; Zhang et al., 2019). However, only a minimal portion of the organic Se synthesized in the roots can be transported to the aboveground parts (Huang et al., 2015; da Silva et al., 2020), leading to a higher accumulation of Se in the roots where it was absorbed by rice plants. This also resulted in a significant increase in the root-to-soil transfer coefficient (TF_Root/Soil_) with straw and straw biochar treatments in this study, but it decreased the transfer coefficient of Se from the roots to the stems and from the stems to the leaves (TF_Stem/Root_) (**Table 3**).

The changes in the status of soil nutrients caused by management measures affected the status of nutrients of plants (**Figure 4**). This was attributed to the fact that returning straw and straw biochar to the field increased the total N, P, and K nutrients in the soil and improved the supply of available nutrients (Meng et al., 2019). Our previous research results have indicated that returning straw and biochar to the field enhances the N transfer capacity of the leaves and the absorption capacity of the grains, promoting the transfer of nutrients to the upper organs of plants (Li et al., 2023), which is consistent with the results of this study. More crucially, the transport of Se within rice plants may be associated with the nutrient contents within the plants (**Figure 6**). The correlation analysis of Se with the N, P, and K contents in rice in this study revealed a significant negative correlation between Se and the content of P and K in the plant (**Figure 6C**). It may be linked to the influence of the P nutritional status on the subcellular distribution and forms of Se, ultimately regulating the transport of Se in rice (Wang et al., 2020). Previous studies have indicated that organic Se uptake via aquaporins and K^+^ channels (Wang et al., 2022a) and the peptide transporters mediated the root-to-shoot translocation (Zhang et al., 2019). The K nutritional status of the rice plant may compete for K^+^ channels and affect the activity of transport proteins (Johnson et al., 2022; Wang et al., 2022a), thereby influencing the absorption and transport of organic Se.

Furthermore, the PLS-PM model analysis revealed that soil chemical properties and the interaction of root nutrients significantly influenced the Se transfer coefficients in various parts of rice, thereby increasing the Se enrichment factor in rice. The absorption of Se in the rice roots and its transport from the leaves to the rice grains were critical processes influencing Se enrichment in rice. Under reducing conditions in paddy fields, Se(IV) was the dominant form of Se in the soil solution, and Se absorbed by rice was mainly in the form of selenite. The transport of selenite to the aboveground parts required the reduction to Se proteins in the roots (da Silva et al., 2020). Therefore, the coupled interaction between N, P, and Se in the roots may be a key factor affecting the transport of Se to the aboveground parts (Wang et al., 2017; Chang et al., 2019; Lei et al., 2022). Moreover, the activation and retranslocation process of Se in rice, from the leaves to the grains through the phloem, was considered a crucial physiological mechanism for Se accumulation in rice grains (Zhang et al., 2017; Zhang and Chu, 2022). Actually, most nutrients were translocated into the seeds from the source leaves through the phloem pathway (Zhang et al., 2007). Wang et al. (2021) observed that leaves played a direct or indirect role in the process of Se accumulation in grains, which was consistent with our results.

## Conclusion

In this study, the impact of straw and straw biochar returning on the transformation of Se forms in Se-rich paddy soils and the absorption and translocation of Se in rice plants were investigated. The experiment results showed that the incorporation of straw and straw biochar into the fields increased the bioavailability of Se in the paddy soil. Moreover, the increased soil-bioavailable Se was primarily attributed to the activation of FMO-Se and OM-Se, which were regulated by changes in soil properties induced by straw and straw biochar incorporation. Additionally, the migration of Se in rice plants was significantly influenced by differences in rice tissues and their nutrients’ interactions. In the soil–rice system of Se-rich paddy fields, the absorption of Se by rice roots and its transportation from the leaves to grains were crucial processes that affected Se enrichment in rice. However, these processes were modulated by soil properties (pH, SOM, AP, and AK) and interactions of root nutrients (N, P, Se) induced by the incorporation of straw and straw biochar. Overall, the management measures to improve the Se content in grain in Se-rich paddy fields should consider the factors affecting the absorption and transport of Se in the soil–rice system and the interaction between Se and other elements in those processes.

## Data Availability

The raw data supporting the conclusions of this article will be made available by the authors, without undue reservation.
