# Supplementary material for: The interaction between selenium and other elements in soil and rice roots shaped by straw and straw biochar regulated the enrichment of selenium in rice grain
Source: Front Plant Sci. 2024 Sep 20;15:1387460. doi: 10.3389/fpls.2024.1387460 (PMC11449701; doi:10.3389/fpls.2024.1387460)
Supplement: Supplementary file 1 [file Table1.docx]

Table S1 Effects of straw and straw biochar returning on the distribution of different forms of Se in soil (%)

| Treatment | SOL-Se | EXC-Se | FMO-Se | OM-Se | RES-Se |
| --- | --- | --- | --- | --- | --- |
| NPK | 0.53±0.03c | 2.04±0.18a | 7.95±0.35a | 32.99±2.85a | 57.02±3.05b |
| NPK+S | 0.98±0.12b | 2.15±0.31a | 6.60±0.43b | 30.73±2.65ab | 60.52±3.32 ab |
| NPK+B | 1.28±0.07a | 2.14±0.34a | 5.59±0.52c | 27.41±2.14b | 64.85±2.59a |

Different letters in the same column in the table indicate significant differences between treatments (*p*<0.05).
